# Dermatitis artefacta

**DOI:** 10.4103/0019-5545.43061

**Published:** 2005

**Authors:** Bimal Tamakuwala, Parag Shah, Kamlesh Dave, Ritambhara Mehta

**Affiliations:** * Junior Resident, Department of Psychiatry, Government Medical College and New Civil Hospital, Surat; ** Junior Resident, Department of Psychiatry, Government Medical College and New Civil Hospital, Surat; *** Assistant Professor, Department of Psychiatry, Government Medical College and New Civil Hospital, Surat; **** Associate Professor, Department of Psychiatry, Government Medical College and New Civil Hospital, Surat

**Keywords:** Dermatitis artefacta, personality factors

## Abstract

Dermatitis artefacta, also known as factitious dermatitis, is a condition in which cutaneous lesions are self-inflicted and are the result or manifestation of some psychological conflicts. This report presents the case of a 20-year-old man, whose initial presentation resembled a dermatological disorder. Psychological and personality factors as well as issues in the management are discussed.

## INTRODUCTION

Dermatitis artefacta is a psychocutaneous disorder in which the skin is the target of self-inflicted injury. Patients intentionally produce lesions to assume the sick role and typically deny the self-inflicted nature of the disorder. The history is often vague. The diagnosis of dermatitis artefacta depends on the doctor having a high index of suspicion.^1^ Except for mild transient cases triggered by an immediate stress, the prognosis for cure is poor. The condition tends to wax and wane with the circumstances in the patient's life.^2^

## THE CASE

A 20-year-old unmarried male was referred to the Psychiatry Department by the Skin Department. He had multiple, well-demarcated skin lesions on the extremities, abdomen, back and face for the past 3 months. The lesions had clear-cut borders with fresh blood oozing from some of them. The patient had an injury on the back of the right foot due to a fall from a bicycle. Reportedly, he took Tab. penicillin for 2 days after which he developed severe itching culminating in rashes and excoriations over both the hands and upper extremities. It gradually progressed to the abdomen, back, lower limbs and face. His father took him to a dermatologist. He was treated with various antibiotics and antihistaminics but showed no improvement.

**Fig. 1:**
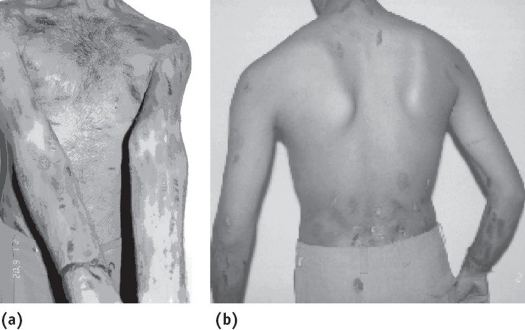
**(a)** The patient with dermatitis artefacta **(b)** In the same patient the back is spared, as the area is difficult to reach with the hands

On presentation, the patient had well-demarcated multiforme skin lesions all over the body except the middle of the back (Fig. 1). Each lesion measured about 2–3 cm in length and 1 cm in width. The patient was anxious and concerned about his skin lesions. He had no hallucinations or delusions. Several interview sessions were conducted with the patient and his parents.

The family history revealed that his father had remained away from the home for occupational reasons when the patient was 2–10 years old. He lived with his grandparents, mother and brothers up to the age of 15 years. The patient described his father as ‘authoritative’; on the other hand, there was over involvement and overprotection by the mother and grandfather. He was made to assume a ‘sick role’. The entire family believed him to be a physically and mentally weak person, from childhood. He had frequent conflicts with his younger brother whom he viewed as a competitor for attention, and who disregarded him as an elder brother.

The patient's birth history was uneventful and he had no major medical illness. His motor, social and cognitive milestones developed at age-appropriate levels. He had studied up to secondary school, after which he left studies and started doing minor farm work at his native village. After coming to the city, where his family had settled down after his father's return, he started working in the printing industry but did not attend his workplace regularly. He frequently changed jobs due to one reason or the other. He was not able to take decisions about his choice of work. Since his adolescence he had not been able to do any work consistently. Sometimes, he became totally submissive to his father's decision and said that he would follow his father's advice; whereas at other times, he became passively rebellious towards his father and did not go to work; he even lied to his parents. Once he went to Mumbai for 10 days without informing anyone. During that time and twice in his village he became physically involved with women. He was beaten up by the village elders and had to leave the village for some time. The patient gave this history without much probing which was corroborated by his parents.

On mental status examination, the patient had feelings of worthlessness and helplessness. He had low self-esteem as he was unable to fulfil familial responsibilities. He was preoccupied with his somatic complaints and repeatedly asked to be cured of the tingling and itching of the skin. He insisted that this was some reaction to the drug which he had taken on his father's advice. He viewed his future as hopeless and had frequent suicidal thoughts. He also felt guilty about past sexual relations. He attributed his inability to work to his skin disease.

The patient's personality was assessed by psychological tests. He had feelings of inadequacy and was hypersensitive to negative evaluation, which was apparent by his avoidance of occupational activities. He also had borderline traits evidenced from the instability of his interpersonal relationships and affect, and disturbed self-image. He used physical symptoms as a defence mechanism to protect his weak ego structure, which made it difficult for him to fulfil social responsibilities. He showed generalized anxiety with depressive affect and a danger of personality disintegration on Thematic Apperception Test (TAT). There were repetitive emotions of worry, concern regarding his health, death due to suicide, accidental reasons and insanity. On the Rorschach test, the patient gave overall underproductive protocol with 3 popular responses—one human response, rejection of father and sex-card—which suggest a certain amount of guardedness and a possible sexual conflict.

The patient was put on 20 mg fluoxetine and anxiolytics along with supportive and insight-oriented psychotherapy and family counselling. He continued under the care of the dermatologist. Within 2–3 months, he showed about 50%–60% improvement. The skin lesions, especially those over the face, had resolved and there were occasional new lesions on the limbs and trunk. After 6 months of follow up, his symptoms are waxing and waning; these can be correlated with his family environment and experience of subjective stress. It is extremely difficult to progress in psychotherapy due to high somatic preoccupation, emerging beliefs regarding drugs and their effect on the body, dependent and passive–aggressive traits, low psychological mindedness and negative counter-transference.

## DISCUSSION

In dermatitis artefacta, the patient creates skin lesions to satisfy an internal psychological need, usually a need to be taken care of. The prevalence is about 0.3% among dermatology patients with the highest frequency during adolescence and young adulthood. An early age of onset and somatic preoccupation are hurdles in the management of this disease. Direct confrontation should be avoided, as it may be counter-productive. Limit-setting for the protection of both the therapist and patient; creation of an accepting, empathic and non-judgemental environment; and close supervision of symptomatic dermatological care permit the development of a therapeutic relationship in which psychological issues may gradually be introduced. It is important to give the patient a gradual orientation to limited responsibilities and to deal with the anger and frustration of the family members during management.

The role of medication is highly debatable and several studies have reported contradictory results.^3^–^6^ Antidepressants can be useful in the presence of depressive symptoms. Selective serotonin reuptake inhibitors (SSRIs) have a better acceptance with lower side-effects.
